# Simultaneous fMRI and EEG during the Multi-Source Interference Task

**DOI:** 10.1371/journal.pone.0114599

**Published:** 2014-12-09

**Authors:** John A. Robertson, Alex W. Thomas, Frank S. Prato, Mikael Johansson, Henrietta Nittby

**Affiliations:** 1 Lawson Health Research Institute, St. Joseph's Health Care, London, Ontario, Canada; 2 Techna Institute, University Health Network, Toronto, Ontario, Canada; 3 Department of Psychology, Lund University, Lund, Sweden; 4 Department of Neurosurgery, Lund University, BMC D10, SE-221 85, Lund, Sweden; University of Vienna, Austria

## Abstract

**Background:**

fMRI and EEG are two non-invasive functional imaging techniques within cognitive neuroscience that have complementary advantages to obtain both temporal and spatial information. The multi-source interference task (MSIT) has been shown to generate robust activations of the dorsal anterior cingulate cortex (dACC) on both a single-subject level and in group averages, in fMRI studies. We have now simultaneously acquired fMRI and EEG during a cognitive interference task.

**Materials and Methods:**

Healthy volunteers were tested in an MRI scanner with simultaneous EEG and fMRI recordings during the MSIT.

**Results:**

The interference condition significantly increased the reaction time in the task. The fMRI analyses revealed activation of dACC as expected, in all subjects at the individual level and in group analyses. The posterior cingulate cortex was de-activated. Simultaneous EEG showed the expected anterior distribution of the interference effect, as it was restricted to frontal sites within a time frame of 80–120 ms post response.

**Conclusion:**

The MSIT task is a reliable task for interference evaluation. fMRI shows robust activation of dACC and by adding EEG, an interference effect can be noticed within a temporal interval of 80–120 ms after the response, as a CRN (correct response negativity). This means that EEG could add a more detailed temporal aspect to the fMRI data from an interference task, and that despite the hostile environment within an MRI scanner, EEG data could be used.

## Introduction

Functional magnetic resonance imaging (fMRI) and electroencephalography (EEG) are two non-invasive functional imaging techniques within cognitive neuroscience. They have complementary advantages in that EEG records millisecond changes in brain electrical but has poor spatial information while fMRI can provide spatial localization of activity within millimeters but is limited in temporal resolution to several seconds. The fMRI information is based upon magnetic susceptibility of the blood during brain activation. With BOLD fMRI (blood-oxygen level dependent functional MRI), changes in the MRI signal arise due to local changes in blood oxygenation, flow, and volume, that result from the metabolism associated with neuronal activity. However, direct relationships with neuro-electric activity cannot be made. With EEG on the other hand, the electrical activity generated by underlying brain structures can be measured at the scalp, with high temporal resolution. With recordings of event-related potentials (ERPs), evaluations of the processing within the brain following specific stimuli can be made. The drawback of EEG is that it provides only limited spatial resolution. Theoretically, combining fMRI and EEG recordings enables the temporal dynamics of information processing to be characterized and linked with spatial information to implicate the involvement of well-defined neural networks [1]. The spatial information from fMRI can aid in the source reconstruction of ERPs recorded at the scalp, improving the understanding of cognitive implementation in the brain.

During cognitive interference tasks, the processing of one stimulus feature impedes the simultaneous processing of a second stimulus attribute [2]. The multi-source interference task (MSIT) compares a cognitive interference task to a control task. It has been shown to generate robust fMRI activations of the dorsal anterior cingulate cortex (dACC) on both a single-subject level and in group averages [2]–[3]. The dACC is involved in decision-making, target detection, novelty detection, error detection, response selection, and stimulus/response competition [2]. In addition to the specific dACC activation during the MSIT, the cingulo-frontal-parietal (CFP) attentive/cognitive network is also activated, which includes daMCC (the dorsal anterior midcingulate cortex, contributing to cognitive processes), DLPFC (the dorsolateral prefrontal cortex, often being co-activated with daMCC during cognitive tasks), the premotor and primary motor cortex (responsible for planning and execution of non-automatic tasks), the inferior temporal gyrus, and the superior parietal lobule [3]. Furthermore, the MSIT has been found to deactivate the perigenual anterior cingulate cortex (pACC), involved in emotional processing [2]–[3].

Regarding EEG, it has been shown that error-related negativities (ERN) are present in the time range of 100 ms after an erroneous response [4]. The ERN is described as a negative potential, which can have a peak amplitude as high as 10 µV and peak around 100–150 ms after the onset of the activity associated with the erroneous response [4]. A similar potential, known as the correct response negativity (CRN), has been associated with the execution of correct responses [4]. A CRN has been reported following correct response trials during a choice reaction time task [5]. The CRN/ERN was focused at FCz, and with the same time course for correct as well as error trials. This has been identified as a response-locked fronto-central negativity. ERNs and CRNs have been proposed to reflect the same functional process, namely response monitoring [6]. We therefore chose to analyse response-locked ERPs, since it would be reasonable to expect a difference in amplitude of CRN in interference versus control, due to different difficulties of the tasks.

With the understanding that the fMRI activations associated with the MSIT task are well-understood, we set out to replicate the main findings described by Bush et al. [2] – dACC activation as measured by BOLD fMRI – in healthy volunteers, and to correlate these fMRI findings to simultaneous EEG recordings.

## Materials and Methods

### Subjects

18 healthy (17 right-handed, 1 left-handed; 10 females, 8 males) participants were tested in this study. The subjects were recruited among students or researchers at the Lawson Health Research Institute or at the University of Western Ontario, London Ontario Canada. All subjects had normal or corrected-to-normal vision, meaning that they could clearly see the numbers displayed during the MSIT. Exclusion criteria included claustrophobia as well as standard MRI exclusion criteria (e.g. cardiac pacemakers, brain aneurysm clips or surgical clips). We controlled for factors that we hypothesized could affect the comparisons between subjects, such as an intake of caffeine, nicotine, and alcohol. No subject had a history of major medical problems, medications (except birth-control pills in 4 cases), major psychiatric illness, major head injury, or neurological disease. The research protocol was approved by the University of Western Ontario Health Sciences Research Ethics Board (London, Ontario, Canada). Subjects were informed that they would wear an EEG cap for EEG registrations, go through a practice version of a cognitive test and thereafter enter the fMRI scanner for simultaneous fMRI and EEG recording while doing the cognitive task twice. The left-handed participant did not report any problems using the right hand.

### The MSIT

Subjects were given an MRI-compatible four-button keypad (NeuroScan, Charlotte, NC) and instructed that the keypad buttons represented one, two and three from left to right (as described in the protocol by Bush et al. [3]); the right-most button was not used. The subjects were told to use their right index, middle and ring finger to respond. They were instructed that three numbers (0, 1, 2, or 3) would appear in the center of the screen every few seconds and that in-between there would be a white marker for fixation. One number – the target number – would always be different from the other two numbers. The subjects were instructed to report, via button-press, the identity of the target number that was different from the other two numbers.

**Figure 1:**
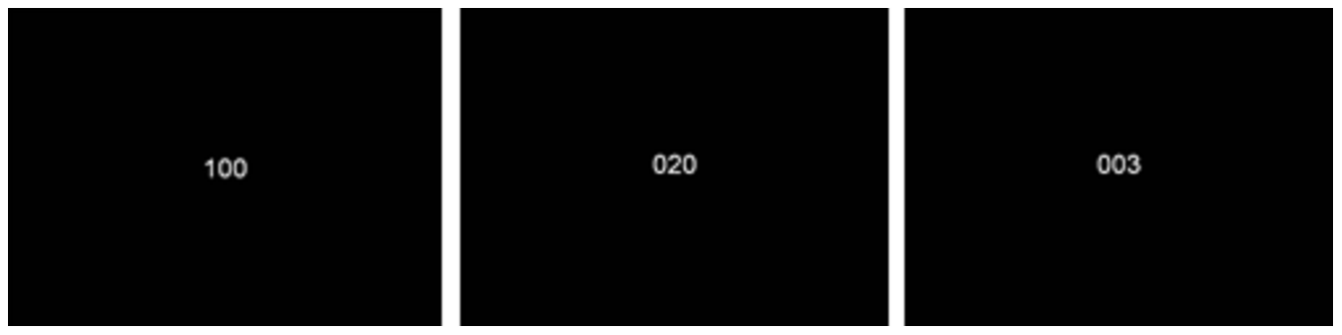
Control task. These three are all possible iterations of the control condition.

**Figure 2:**
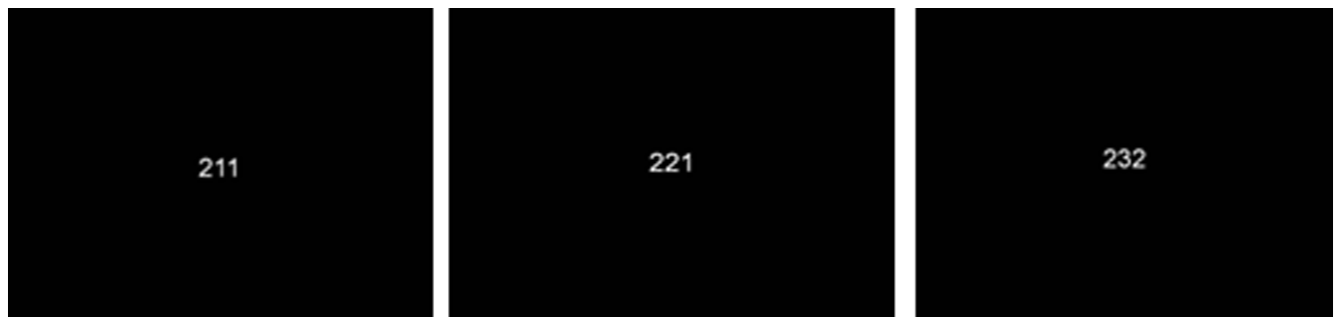
Interference task. There are many more possible iterations of the interference task.

During the control tasks, the target number always matched its position and was accompanied by two zeros in the two other positions (see Fig. 1). In the interference tasks on the other hand, the target number never matched its position, and no zeros would be included; instead the distracters themselves would be potential targets (see Fig. 2). It was emphasized that the subjects should report the target number regardless of its position and that the subjects should answer as quickly as possible, but not sacrifice accuracy for speed.

After the instructions were reviewed and prior to entering the MRI scanner, the subjects completed a 5 min long practice version of the task. Once in the MRI scanner, the MRI-compatible keypad was placed next to the subject on their right side and the subject was reminded to press hard enough on the buttons, to keep their eyes fixed upon the white marker and try to lie as still as possible. MSIT stimuli were generated by the NeuroScan software (Stim2, NeuroScan, Compumedics Limited, Australia) and projected onto a screen situated at the rear of the magnet, which the subjects could see in a mirror attached to the MRI headcoil; the projection and mirrors were arranged so that numbers appeared right-side-up. The MSIT was run in tasks of control and interference stimuli. The tasks, of which each subject completed two, were randomized within each task, with a 500 ms stimulus duration and an inter-task interval (ITI) of 1,750 ms. Each stimulus was followed by a white marker for 1,250 ms before the next stimulus was presented. Four tasks of control tasks were alternated with four interference tasks, the order of presentation of the control and interference tasks was fixed (FCICICICIF; F stands for the fixation period initiating and finalizing each test, C =  control, I =  interference). Thus the subjects completed 24 tasks during each task (as recommended by Bush et al. [3]). Reaction time (RT) and accuracy of performance were captured by the Stim2 software for analysis. The test was performed twice.

### fMRI Procedure

After obtaining informed consent, subjects were instructed about the MSIT task and were fitted into an MRI-compatible EEG cap. The subjects were placed in the Siemens Verio 3.0 T MRI scanner (Siemens, Erlangen Germany) with the Siemens 12-channel phased array head coil; foam stabilizers were placed around the head of each subject in order to minimize head movement. With the EEG cap on, each participant completed a 30 minute fMRI session. The session began with a localizer scan (13 seconds) for BOLD and anatomical acquisition placement. After this the two task repetitions were acquired with BOLD fMRI (blood oxygen level dependent functional MRI, TR = 3000 ms, TE = 30 ms, matrix size  = 64×64, voxel size was 3.2×3.2×3.2 mm, 30 abutting slices, single-shot EPIs, flip  = 90°) and EEG while the subject performed the MSIT (6 minutes and 42 seconds for each MSIT). Between the first and second MSIT scans, high-resolution T1 anatomical images were acquired with a 3D FLASH (fast low angle shot, TR = 19 ms, TE = 4.92 ms, matrix  = 256×256, 160 images) sequence for co-registration of the functional images.

### fMRI Analyses

Brain Voyager QX 2.1.0.1532 (Brain Innovation, Maastricht, The Netherlands) was used to analyze the functional images associated with the MSIT [7]. Control versus interference comparison images were produced for each experimental subject as well as on a group level. The trials were blocked, and therefore the difference in RT was not modelled. All images are presented in the radiological convention (left-is-right). Individual datasets were pre-processed with slice scan time correction, three-dimensional motion correction (trilinear interpolation) and temporal filtering (high-pass filter). Functional slice-based data were aligned with the three dimensional anatomical images, and for group analyses the brain activity was related to a common anatomical space through a Talairach transformation. Interference vs control was used as the functional task, with the fixation period excluded from the design as a predictor of no interest; the predictors were convolved with the canonical hemodynamic response function using Brain Voyager's default settings. A general linear model analysis was performed as a multi-subject analysis and 1 cm^3^ ROIs from the centres of significant activation clusters (with a Bonferroni corrected p-value <0.05) were defined. The Bonferroni correction was based on the number of voxels in the comparison, not the number of ROIs.

### Electrophysiological Measures

EEG was recorded using a 64 electrode fMRI-compatible EEG cap (MagLink Cap, Neuromedical supplies, Neuroscan) with Ag/AgCl electrodes placed according to the international 10/20 electrode placement standard. A scalp-brushing technique was used prior to cap placement to improve impedences, and electrolytic gel was introduced into the electrode cavity. The ground electrodes were located between the 10/20 positions of FCZ and FZ and the reference located between the 10/20 positions of CZ and CPZ in the 64 electrode cap. A bipolar electrode pair located above and below the right eye recorded ocular activity (electro-oculogram, EOG). The electro-cardiogram (ECG) was monitored with bipolar electrodes located above the second intercostal space (I2) on the left side of the chest at either side of the heart, in order to model and remove ballistocardiogram activity. A pulse oximeter was also attached to the subjects' left index fingers in the MRI. Before entering the fMRI scanner, electrode positions, physical landmarks and head shape were digitized using 3D SpaceDx (Neuroscan SCAN). Electrodes in the MagLink cap were connected to EEG amplifiers (SynAmps2, Neuroscan) (a 70 Channel amplifier system, consisting of 64 monopolar, 4 bipolar and 2 high-level channels) via the carbon-filter conductor cable through RF filters in a waveguide in the walls connecting to the fMRI room and led further on to the data acquisition system. Data was sampled at 1,000 Hz. Data was stored and analyzed offline (using Neuroscan).

Pre-processing was done with Neuroscan Edit 4.3 and comprised steps to remove pulse sequence artifacts and ballistocardiogram artifacts, and to compensate for DC drift through off-line filtering of the data (bandpass with high-pass cut-off at 0.5 Hz and low-pass cut-off at 30 Hz, 24dB). The continuous EEG was re-referenced to averaged mastoids and epoched in a response-locked fashion from −400 to 700 ms around the time of the response. The pre-response interval was used for baseline correction. Epochs containing recording artefacts or erroneous responses were excluded from further analyses. ERP averages were formed separately for interference and control tasks.

ERP waveforms were quantified by measuring the mean amplitudes in the 80–120 ms time window at frontal (F1, Fz, F2), central (C1, Cz, C2), and parietal (P1, Pz, P2) sites. The time window and electrodes were selected based on previous literature and a visual inspection of the current data, and aimed at tapping the frontocentral post-response negativity. Erroneous responses, i.e. when the subjects chose the wrong answer in the control or interference task, were removed, after which the recordings were baseline corrected and fitted to a common average. EEG data was successfully acquired from 17 subjects (for one subject the artifact reduction could not be done properly due to too many artifacts). Bad electrodes were disregarded (an electrode was considered to be bad if the recordings were too noisy due to high impedance values in 1 or more of the 17 subjects), as identified by visual inspection of the EEG. Ballistocardiogram artifact reduction was performed using the ECG electrode, when this electrode functioned properly (in 7 subjects). For the subjects where ballistocardiogram artifact reduction could not be completed with ECG electrode data, the pulse oximeter data was used.

## Results

### Behavioral Results

The reaction time (RT) was significantly increased in the interference tasks as compared to the control tasks (p = 1.6×10^−23^, two-tailed t-test). The median interference effect, regarded as RTinterference-RTcontrol, was 250 ms (SD 60 ms). RT for erroneous responses was discarded. The combined accuracy for the 18 included subjects was 86% in the control tasks and 84% in the interference tasks. The number of mistakes in each task of 24 tasks was as a median 1.25 (SD 4.3) in the control group as compared to 1.5 (SD 4.3) in the interference group (2-sided t-test p = 0.04).

### fMRI Analyses

**Figure 3:**
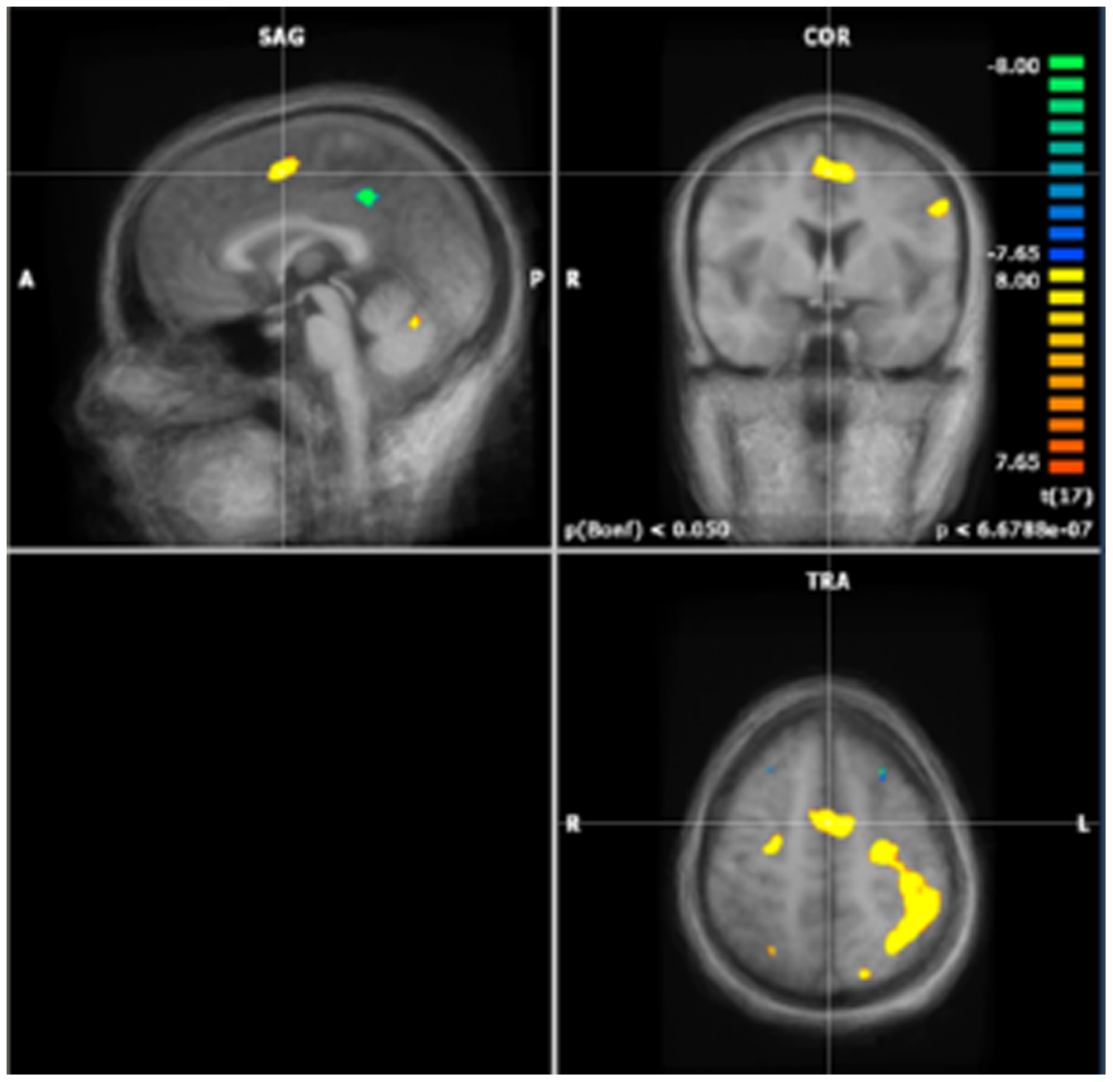
Activation of the dACC, as defined when the fMRI scans of all individual subjects are analyzed as a group-analysis.

Data from 18 subjects could be utilized for fMRI analyses, but for 1 subject, half of the data was lost due to recording difficulties. Performing a ROI analyses, a list of 17 regions were found to have a significantly altered expression in the control situation as compared to the interference situation (Bonferroni corrected to p<0.05 (uncorrected p<6.67×10^−7^)) (Table 1). Twelve regions were activated during the interference task as compared to the control task, and five were deactivated in the interference task as compared to the control task. Among the activated regions, the dACC could be identified (Talairach coordinates 0,0,50) (Fig. 3), and also at a single subject level, it was found that the dACC was activated in all subjects during the interference as compared to the control situation. The posterior cingulate cortex was de-activated (Talairach co-ordinates −3,39,39). There was increased activation both in the left and right parietal lobe (Talairach co-ordinates −43, −39, 45 and 26, −53, 42 respectively) in interference as compared to control. Increased activation with the interference condition was also seen in the left and right motor cortex (Talairach co-ordinates −28, −14, 54 and 28, −12, 53 respectively).

**Table 1:** All 17 ROIs as defined by the fMRI analysis.

ROI	Talairach Coordinates	Region name	Region Activated or Deactivated
1	0,0,50	dACC	Activated
2	−3, 39, 39	Posterior cingulate	Deactivated
3	−43, −39, 45	left parietal lobe	Activated
4	26, −53, 42	right parietal lobe	Activated
5	−28, −14, 54	left sensory motor cortex	Activated
6	28, −12, 53	right motor cortex	Activated
7	−27, 22, 52	Frontal cortex	Deactivated
8	22, 19, 53	Frontal cortex	Deactivated
9	−50, −2, 33	Frontal cortex	Activated
10	45, −66, 29	right parietal lobe	Deactivated
11	28, 18, 13	right insula	Activated
12	−13, −20, 12	left thalamus	Activated
13	19, −35, −10	right hippocampus	Deactivated
14	−44, −66, −16	left occipital lobe	Activated
15	−1, −62, −21	Cerebellum	Activated
16	28, −44, −27	Cerebellum	Activated
17	−34, −51, −28	Cerebellum	Activated

The interference effect was calculated by subtracting RT(interference) – RT(control) for each subject; this was correlated to the fMRI data for each subject and ROI. The Pearson's r correlation coefficient between interference effect and accuracy in the interference situation was 0.48. There was no strong correlation between interference effect and fMRI data for each subject (Pearson's correlation coefficient −0.5<r<0.5 except for ROI 14 where r = 0.54).

There was no strong correlation between the interference effect (RTinterference-RTcontrol) and the accuracy in the control or interference tasks. When correlating the accuracy in the interference task to the activation in the ROIs, the Pearson's r coefficient was −0.5<r<0.5 for all ROIs expect ROIS 12 (r = 0.54).

### EEG Analyses

**Figure 4:**
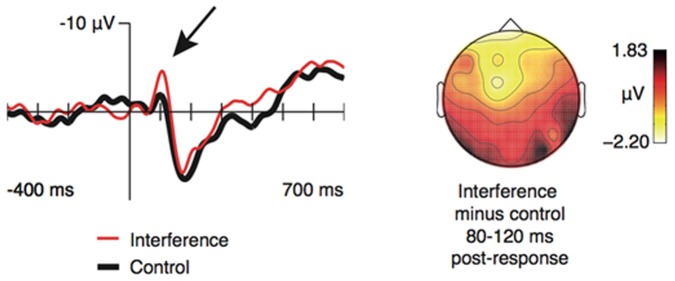
EEG analyses of interference versus control, with a generally more negative amplitude in the interference condition as compared to the control condition. ERP for Fz electrode shown.

A repeated measures ANOVA with the factors Conflict (interference vs control), Anterior/posterior (frontal, central, parietal), and Hemisphere (left, mid, right) revealed a significant main effect of Conflict, F(1,16)  = 6.035, p = .026, η2 = .274, which was due to generally more negative amplitude in the interference condition as compared with the control condition (see Fig. 4). A significant interaction between Conflict and Anterior/posterior, F(2,32)  = 5.156, p = .011, η2 = .244, indicated further that the difference between experimental conditions varied as a function of scalp position. Follow-up analyses showed the expected anterior distribution of the effect, as it was restricted to frontal sites, F(1,16)  = 11.769, p = .003, η2 = .424 (marginally significant at central, F(1,16)  = 3.592, p = .076, η2 = .183, and parietal sites, F(1,16)  = 3.604, p = .076, η2 = .184). Neither interaction between conflict and hemisphere nor the three-way interaction between conflict, anterior-posterior, hemisphere were significant (n.s.).

To establish whether there is a functional relation between ERP CRN and the activations seen upon fMRI, an analysis with correlation the beta values from the ROIs for each individual subject to the ERPs generated in the time window of 80–120 ms for each individual subject was performed. Correlation of the beta values from the ROIs for each individual subject to the ERPs generated in the time window of 80–120 ms for each individual subject was analysed. To increase the power, frontal (F1, Fz, F2), central (C1, Cz, C2), and parietal (P1, Pz, P2) sites were analyzed as three different categories. We found no correlation between the ROIs and the ERPs in the frontal, parietal or central sites (N.S).

## Discussion

In this study of simultaneous fMRI and EEG during the MSIT, we could detect an effect of the interference condition, with reduced accuracy and increased reaction time during the interference tasks as compared to the control tasks, as expected from previous studies [3]. Even with the distraction inherent to the MRI environment, these behavioral effects were robust and detectable with a relatively small sample group size and short task duration.

With our fMRI analysis we found an activation of the dACC in the interference group as compared to the control group, both at a group-level and single-subject level, whereas the posterior cingulate cortex was de-activated. Both left and right parietal cortices were activated. Also, in the left and right motor cortex activation could be detected, areas responsible for the execution of non-automatic tasks. Furthermore, the occipital lobe was activated during interference as compared to control, which might be due to increased ocular impressions in order to register the interference information. The simultaneous EEG registration revealed a significant effect of conflict, with anterior distribution restricted to frontal sites within a time frame of 80–120 ms post response, as shown in Fig. 4. This was seen as a CRN focused at FCz, peaking at about 100 ms. Difference in amplitude of the CRN in interference vs control, due to different difficulties of the tasks, could be detected.

We found a concordance between reaction time and accuracy effects, fMRI findings of increased activation in the dACC with the interference condition, and EEG findings of an effect of conflict with frontal distribution of the effect within a time frame of 80–120 ms post response.

Our multimodality study confirms that the interference task produces detectable effects in behavioral, fMRI, and EEG measures that are consistent with previous studies. The MSIT task has been suggested for studies of normal cognition and drug effects for its robust activation. The MSIT task has already been used in studies of people with ADHD, both to map hypofunction in daMCC [8] and in dACC [9] and to document the therapeutic effects of methylphenidate [8]. In a subset of patients with schizophrenia, the dACC was not activated during MSIT cognitive interference, indicating that the activation from the task does differ in certain disease states [10]–[11]. The MSIT task was also used as an attention-demanding task to demonstrate similar brain activations during cognitive demands and pain processing (inferior frontal, superior parietal, premotor and anterior insula cortices) [12].

Despite the previous use of EEG and fMRI, not many studies have focused upon their simultaneous use. Simultaneous fMRI and EEG recording is a powerful tool, increasing temporal and spatial resolution. However, the hostile environment in the fMRI scanner produces huge gradient and pulse artifacts in the EEG, which have to be removed through labourous post-processing. Also, the EEG recording equipment can impair the quality of the fMRI. Previous studies with EEG and fMRI include Formaggio et al. [13], who measured BOLD signals and ERS/ERD during simultaneous fMRI and EEG during finger movement, and found a significant correlation between the positive-negative ratio of BOLD signal peaks and ERD values in the electrodes over the region of activation. Another study was presented by Hesselmann et al. [14], who registered fMRI and EEG simultaneously in a group of 12 subjects performing two tasks shortly after one another, thus investigating the psychological refractory period, meaning that the central processing of the second task is delayed due to limitations in multi-tasking. It was found that the P3 (the post perceptual) potential was delayed during the second task, and that on the BOLD scanning, signals in two bilateral regions in the inferior parietal lobe and precentral gyrus significantly covaried with P3 related activity.

For future studies of cognition and the effects of disease, drugs, or other interventions, the MSIT interference task should be considered as an experimental model. It is simple to set up – requiring minimal equipment – and simple to teach to research participants, it lends itself to uncomplicated fMRI and EEG analysis schemes, and produces robust behavioural, fMRI, and EEG measures.
